# Alterations of Iron Level in the Bilateral Basal Ganglia Region in Patients With Middle Cerebral Artery Occlusion

**DOI:** 10.3389/fnins.2020.608058

**Published:** 2021-01-21

**Authors:** Lei Du, Zifang Zhao, Xiuxiu Liu, Yue Chen, Wenwen Gao, Yige Wang, Jian Liu, Bing Liu, Guolin Ma

**Affiliations:** ^1^Department of Radiology, China-Japan Friendship Hospital, Beijing, China; ^2^Graduate School of Peking Union Medical College, Peking Union Medical College, Chinese Academy of Medical Sciences, Beijing, China; ^3^Department of Anesthesiology, Peking University First Hospital, Peking University, Beijing, China; ^4^Department of Ultrasound Diagnosis, China-Japan Friendship Hospital, Beijing, China

**Keywords:** middle cerebral artery occlusion, quantitative susceptibility mapping, iron, susceptibility, basal ganglia region

## Abstract

**Background and Purpose:** The purpose of this study was to explore the changes of iron level using quantitative susceptibility mapping (QSM) in the bilateral basal ganglia region in middle cerebral artery occlusion (MCAO) patients with long-term ischemia.

**Methods:** Twenty-seven healthy controls and nine patients with MCAO were recruited, and their QSM images were obtained. The bilateral caudate nucleus (Cd), putamen (Pt), and globus pallidus (Gp) were selected as the regions of interest (ROIs). Susceptibility values of bilateral ROIs were calculated and compared between the affected side and unaffected side in patients with MCAO and between patients with MCAO and healthy controls. In addition, receiver operating characteristic (ROC) curves were performed to evaluate the diagnostic capability of susceptibility values in differentiating healthy controls and patients with MCAO by the area under the curve (AUC).

**Results:** The susceptibility values of bilateral Cd were asymmetric in healthy controls; however, this asymmetry disappeared in patients with MCAO. In addition, compared with healthy controls, the average susceptibility values of the bilateral Pt in patients with MCAO were increased (*P* < 0.05), and the average susceptibility value of the bilateral Gp was decreased *(P* < 0.05). ROC curves showed that the susceptibility values of the Pt and Gp had a larger AUC (AUC = 0.700 and 0.889, respectively).

**Conclusion:** As measured by QSM, the iron levels of the bilateral basal ganglia region were significantly changed in patients with MCAO. Iron dyshomeostasis in the basal ganglia region might be involved in the pathophysiological process of middle cerebral artery stenosis and occlusion. These findings may provide a novel insight to profoundly address the pathophysiological mechanisms of MCAO.

## Introduction

It is well-established that stroke has been one of the most common cerebrovascular diseases that could generate neuronal deaths and subsequent disability and mortality. According to pathological origins, stroke can be divided into two forms, ischemic and hemorrhagic stroke, and ischemic stroke accounts for the majority of patients (Mohan et al., 2009). Notably, middle cerebral artery stenosis and occlusion is the most frequent ischemic subtype in clinical practice and the most widely selected experimental model to elucidate the pathophysiology of cerebrovascular ischemic lesions. Although non-infectious neuroinflammation, oxidative stress, and metabolic disturbances of metal ions have been implicated in the process of middle cerebral artery occlusion (MCAO) (Fujioka et al., 2003; Kamel and Iadecola, 2012), the pathogenesis of MCAO has not been fully elucidated.

Iron, mainly stored in the form of ferritin or hemosiderin in brain tissue, is indispensable to the maintenance of normal brain function as it is an important cofactor for various enzymes involved in neurotransmitter synthesis, oxygen transport, electron transfer, and myelin generation (Connor et al., 2001; Munoz and Humeres, 2012). Regardless of the crucial roles in normal physiological functions, overloading of iron in the brain might trigger an abnormal release of toxic free radicals and consequent oxidative damage (Bolt and Marchan, 2010; Kell, 2010). Consistently, recent studies indicated that excessive iron deposition in brain tissue was closely linked to a variety of neurodegenerative diseases, such as Parkinson's disease (PD), Alzheimer's disease (AD), and stroke, which validated the underlying role of brain iron in the pathogenesis and progression of cognitive symptoms (Bishop et al., 2011; Acosta-Cabronero et al., 2013; Murakami et al., 2015; Du et al., 2016).

Compared with the traditional iron measurement techniques, susceptibility weighted imaging (SWI) (Schafer et al., 2009; Shmueli et al., 2009) and quantitative susceptibility mapping (QSM) can non-invasively and quantitatively detect the magnetic susceptibility values of brain tissue with higher specificity and sensitivity. Previous studies had found that the susceptibility value of tissue acquired by QSM was positively correlated with the iron level in deep gray matter nuclei (Langkammer et al., 2012; Deistung et al., 2013; Lim et al., 2013; Sun et al., 2015), which further verified the specific advantage in the measurements of tissue iron content. Currently, QSM has been increasingly used for the diagnosis, grading, and severity prediction of neurodegenerative diseases, both in clinical practice and experimental investigation.

The blood and oxygen in the basal ganglia regions are mostly supplied by the middle cerebral artery, and only a small part is supplied by the anterior cerebral artery and the choroid artery. Ischemia induced by MCAO could cause a variety of pathophysiological complications and subsequently result in brain function disorders and brain damage. Interestingly, the ischemic damages were found not only in the regions supplied by the MCA but also in deep regions such as the basal ganglia region, hippocampus, hypothalamus, and thalamus (Fujioka et al., 2003; El Amki et al., 2015; Pang et al., 2015). A possible explanation is the concomitant occlusions of deep and small cerebral arteries arising directly from the internal carotid artery, at the proximity of the origin of MCA (El Amki et al., 2015). Moreover, several studies have found that long-term ischemia could result in oxidative stress, non-infectious neuroinflammation, acidosis, and metabolic disturbances of metal ions such as calcium (Siesjo, 1988; Kamel and Iadecola, 2012; Yushmanov et al., 2013; Liu et al., 2014). However, it remains unknown whether the metabolism of iron in the basal ganglia regions would change after a long-term ischemia. We speculated that the iron level in the basal ganglia regions may change along with ischemic brain damage secondary to MCAO. The purpose of this study was to explore the changes of iron level using QSM in the bilateral basal ganglia region in MCAO patients with long-term ischemia.

## Materials and Methods

### Subjects

This study protocol was approved by the Ethics Committee of China-Japan Friendship Hospital and informed consent of all the participants was obtained. The severity of stroke of all the participants was measured by the NIH Stroke Scale (NIHSS) scale. A total of 27 healthy controls and 9 adult patients with MCAO were enrolled in this research. The patients with MCAO visited the Department of Neurology of China-Japan Friendship Hospital between March 2018 and March 2019. The diagnosis of MCAO was made by neurological physicians based on clinical symptoms, CT results, conventional MRI, and MR angiography. Patients with MCAO were selected if they met the following criteria: (a) the MR image has no artifacts; (b) the clinical information of the participants is complete and the participants had no other brain diseases, such as tumors or dementia; and (c) the participants had no visual and hearing impairment, capable of coordinating with the completion of scale and MRI examination. There were five cases of left middle cerebral artery occlusion and four cases of right middle cerebral artery occlusion in the patient group. There were five males and four females, with an average age of 61.7 ± 12.3 years (38–79 years). All patients had chronic ischemia and were right-handed. Table 1 shows the detailed clinical information of the nine patients with MCAO.

**Table 1:** Detailed clinical information of nine patients with MCAO.

**Patient**	**Sex**	**Age**	**Affected side**	**Smoke**	**Drink**	**Hypertension**	**Diabetes**	**Handedness**	**Duration (years)**
1	M	38	Right	Y	N	Y	N	Right	2
2	M	61	Left	N	Y	N	N	Right	5
3	F	57	Left	N	N	Y	N	Right	6
4	F	64	Right	N	N	Y	N	Right	2
5	M	50	Left	Y	Y	Y	N	Right	1
6	F	79	Left	N	N	N	N	Right	7
7	F	71	Right	N	N	Y	N	Right	3
8	M	63	Left	N	N	Y	N	Right	4
9	M	72	Right	N	N	Y	Y	Right	6

*MCAO, middle cerebral artery occlusion; M, male; F, female; Y, yes; N, no*.

Healthy controls were recruited from the local communities. Inclusion criteria were as follows: (a) ages range 54–79 (including 54 and 7 years); (b) being right-handed; (c) the patient has no basic diseases such as hypertension and diabetes, no cognitive impairment, and no family history of neurological and psychiatric illness; and (d) MRI examination reveals only small lacunar infarcts. Healthy controls suffering from cardiovascular, neurologic, metabolic, and psychiatric disorders or brain abnormalities were excluded from this study. Finally, 27 healthy controls were enrolled in the present study, 9 males and 18 females, with an average age of 65.1 ± 7.6 years (54–79 years). Table 2 shows all the subjects' clinical information. All subjects received T1-weighted imaging, T2-weighted imaging, 3D T1-weighted imaging, and quantitative susceptibility mapping.

**Table 2:** Demographic data of healthy controls and patients with MCAO.

	**Healthy**	**MCAO**	***P*-values**
No.	27	9	–
Sex (male/female)	9/18	5/4	*P* > 0.05
Age (years)	65.1 ± 7.6 (54–79)	61.7 ± 12.3 (38–79)	*P* > 0.05
Educations	10.4 ± 3.8	–	–
Duration (years)	–	4.0 ± 2.1 (1–7)	–
Handedness (right)	27	9	*P* > 0.05

–*, not significant or not measured; MCAO, middle cerebral artery occlusion*.

### MR Imaging Protocol

A 3.0-T MRI scanner (GE Healthcare, Discovery MR750, Milwaukee, USA) equipped with an eight-channel head coil was used to acquire the data. All participants underwent a T2-weighted imaging scan to exclude craniocerebral organic diseases.

A 3D gradient-echo (GRE) sequence was used for quantitative susceptibility mapping. The parameters were field of view (FOV) = 256 × 256 mm, matrix size = 256 × 256, slice thickness = 1.0 mm, echo time (TE) = 3.2 ms, repetition time (TR) = 22.9 ms, flip angle (FA) = 12°, and scan time = 4 min 24 s.

A 3D T1WI structure image was reconstructed using three-dimensional fast spoiled gradient-echo sequences (3D FSPGR): FOV = 256 × 256 mm, matrix size = 256 × 256, slice thickness = 1.0 mm, TR = 6.7 ms, TE = min full, FA = 12°, number of slices = 192, and scan time = 4 min 10 s.

### QSM

We performed several processing steps to calculate the quantitative susceptibility mapping from the obtained MR phase images. Firstly, a Laplacian-based phase unwrapping was used to perform the phase unwrapping images. Then, we acquired a brain mask using skull-stripping the GRE magnitude image obtained at a TE of 10 ms. Secondly, the phase unwrapping images were divided by 2π^*^TE to get images of the frequency shift in hertz for each echo. Thirdly, background fields were removed with the variable spheric kernel size sophisticated harmonic artifact decrease for the phase-data (V-SHARP) method with a regularization parameter of 0.05 and a maximum radius of 4 mm. Compared with single-echo reconstruction, we averaged the resulting images of all five echoes to acquire a better signal-to-noise ratio after elimination of background fields. Finally, inverse dipole calculations using a least squares and QR factorization-based minimization was performed to get the QSM imaging.

### Image Segmentation

**Figure 1:**
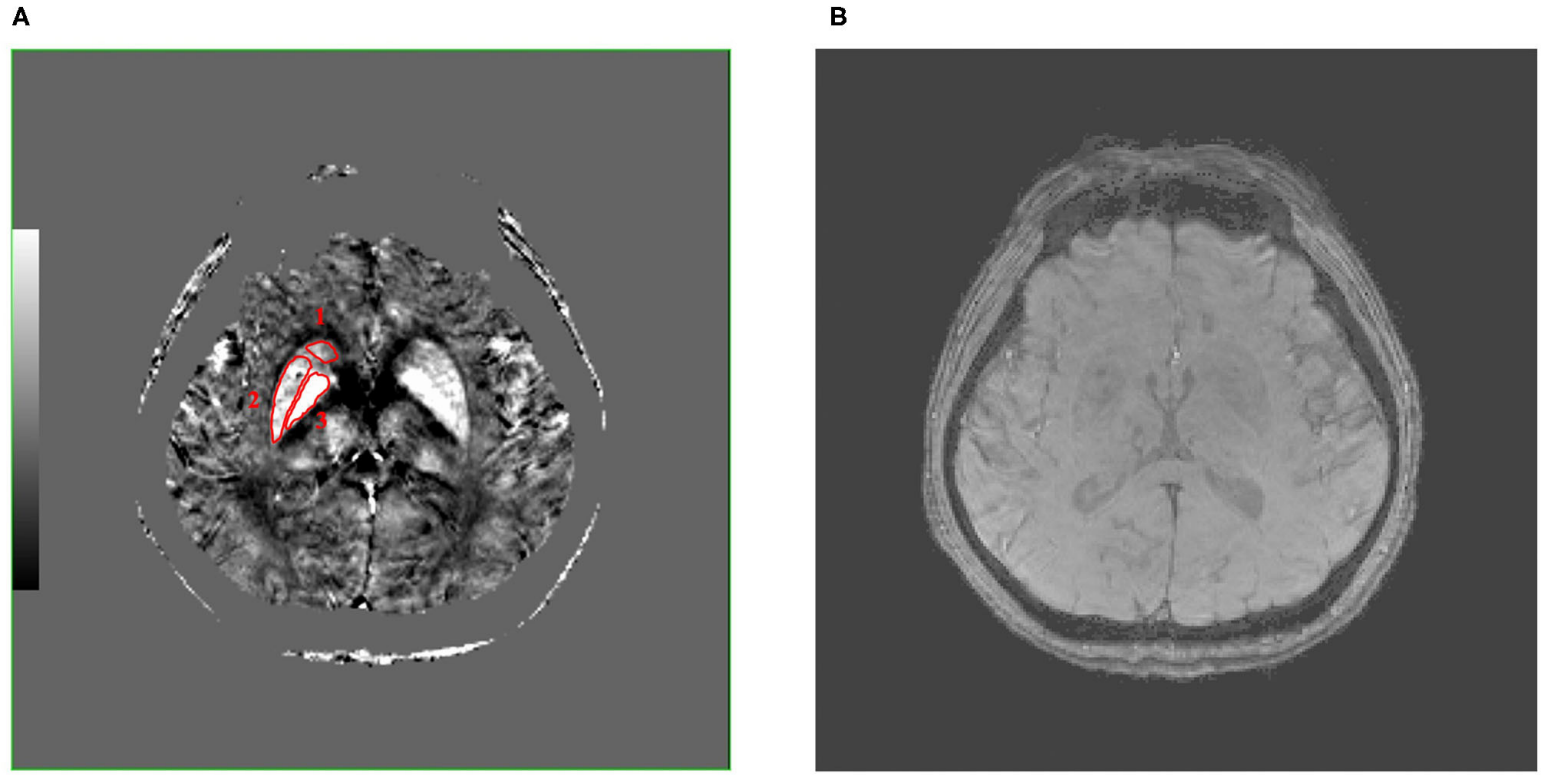
These are QSM **(A)** and SWI **(B)** images of a 50-year-old male patient with MCAO at the same level, and the caudate nucleus (Cd), putamen (Pt), and globus pallidus (Gp) (**number 1–3, separately**) were selected as regions of interest (ROIs), which were encircled in red line in QSM image. Compared with SWI image, QSM image can show the boundary of the Cd, Pt, and Gp more clearly. QSM, quantitative susceptibility mapping; SWI, susceptibility weighted imaging; MCAO, middle cerebral artery occlusion; ROIs, regions of interest.

In the present study, the caudate nucleus (Cd), putamen (Pt), and globus pallidus (Gp) were selected as the regions of interest (ROIs) (Figure 1A). Firstly, the GRE quantitative susceptibility mapping was co-registered to the 3D T1WI, which is to better define the boundaries of each gray nucleus. The ROIs were then manually segmented by two radiologists (Lei Du, 5 years of working experience; Yue Chen, 2 years of working experience) slice by slice using the FuncTool on a GE AW4.6 workstation (GE Healthcare, Milwaukee, WI). They were blinded to clinical and MR imaging information. The susceptibility value and size of three successive slices were acquired in each ROI to eliminate the effects of the occasional case. Also, the average susceptibility values of each ROI were calculated. The unit of susceptibility value was parts per million (ppm).

### Statistical Analysis

Between the patient group and the healthy control group, age was compared using two-sample *t*-test, and gender and handedness were compared using the chi-square (χ^2^) test. The data of age and education was shown in the form of mean ± SD.

The interobserver agreement of two observers was assessed using the intraclass correlation coefficient (ICC), and ICC <0.40 is poor, 0.40–0.59 is fair, 0.60–0.74 is good, and >0.74 is excellent (Oppo et al., 1998).

The paired *t*-test was used to assess the differences of susceptibility values between the left and right hemispheres in healthy controls and between the affected side and the unaffected side in patients with MCAO. Comparisons between patients with MCAO and the healthy controls were investigated using two-sample *t*-test. Moreover, receiver operating characteristic (ROC) curves were performed to evaluate the diagnostic capability of susceptibility values in differentiating patients with MCAO and healthy controls by the area under the curve (AUC) *P* < 0.05 was considered to be statistically significant.

## Results

### Characteristics of All the Participants

The demographic information of all the participants is summarized in Table 2. Finally, 9 MCAO patients (5 males and 4 females, mean age 61.7 ± 12.3 years, range 38–79 years) and 27 healthy controls (9 males and 18 females, mean age 65.1 ± 7.6 years, range 54–79 years, education 10.4 ± 3.8 years) were enrolled in this study. All participants are right-handed. There were five cases of left middle cerebral artery occlusion and four cases of right middle cerebral artery occlusion in the patient group. Then, the left and right gray nuclei in each ROI were divided into the affected side group and the unaffected side group in patients with MCAO. From Table 2, we knew that gender, age, and handedness between the patient group and healthy control group are matched (*P* > 0.05). The interobserver variability was assessed by ICC, and a good agreement was acquired between two radiologists for the susceptibility values (Table 3).

**Table 3:** Intraclass correlation coefficient (ICC) of bilateral caudate nucleus (Cd), putamen (Pt), and globus pallidus (Gp) in patients with MCAO and healthy controls.

	**ROIs**	**ICC of the left hemisphere (95% CI)**	**ICC of the right hemisphere (95% CI)**
MCAO	Cd	0.973 (0.881 to 0.994)	0.448 (−1.446 to 0.876)
	Pt	0.987 (0.943 to 0.997)	0.313 (−2.047 to 0.845)
	Gp	0.877 (0.455 to 0.972)	0.916 (0.628 to 0.981)
Healthy controls	Cd	0.259 (−1.309 to 0.762)	0.742 (0.196 to 0.917)
	Pt	0.706 (0.085 to 0.906)	0.555 (−0.388 to 0.857)
	Gp	0.776 (0.303 to 0.928)	0.796 (0.364 to 0.934)

*ICC, intraclass correlation coefficient; Cd, caudate nucleus; Pt, putamen; Gp, globus pallidus; MCAO, middle cerebral artery occlusion; ROIs, regions of interest; CI, confidence interval*.

The QSM image showed the location of the bilateral Cd, Pt, and Gp in a 50-year-old male patient with MCAO (Figure 1A). Figure 1A shows that there were significant differences between the ROI and surrounding brain regions as seen by the naked eyes, indicating that the iron level in the Cd, Pt, and Gp is more than that in the surrounding brain region. Figure 1B displays a SWI image at the same level as QSM. Compared with the SWI image, the QSM image could show the boundary of the Cd, Pt, and Gp more clearly.

### Comparisons of Susceptibility Values Between the Affected Side and Unaffected Side in Patients With MCAO and Between Left and Right in Healthy Controls

**Figure 2:**
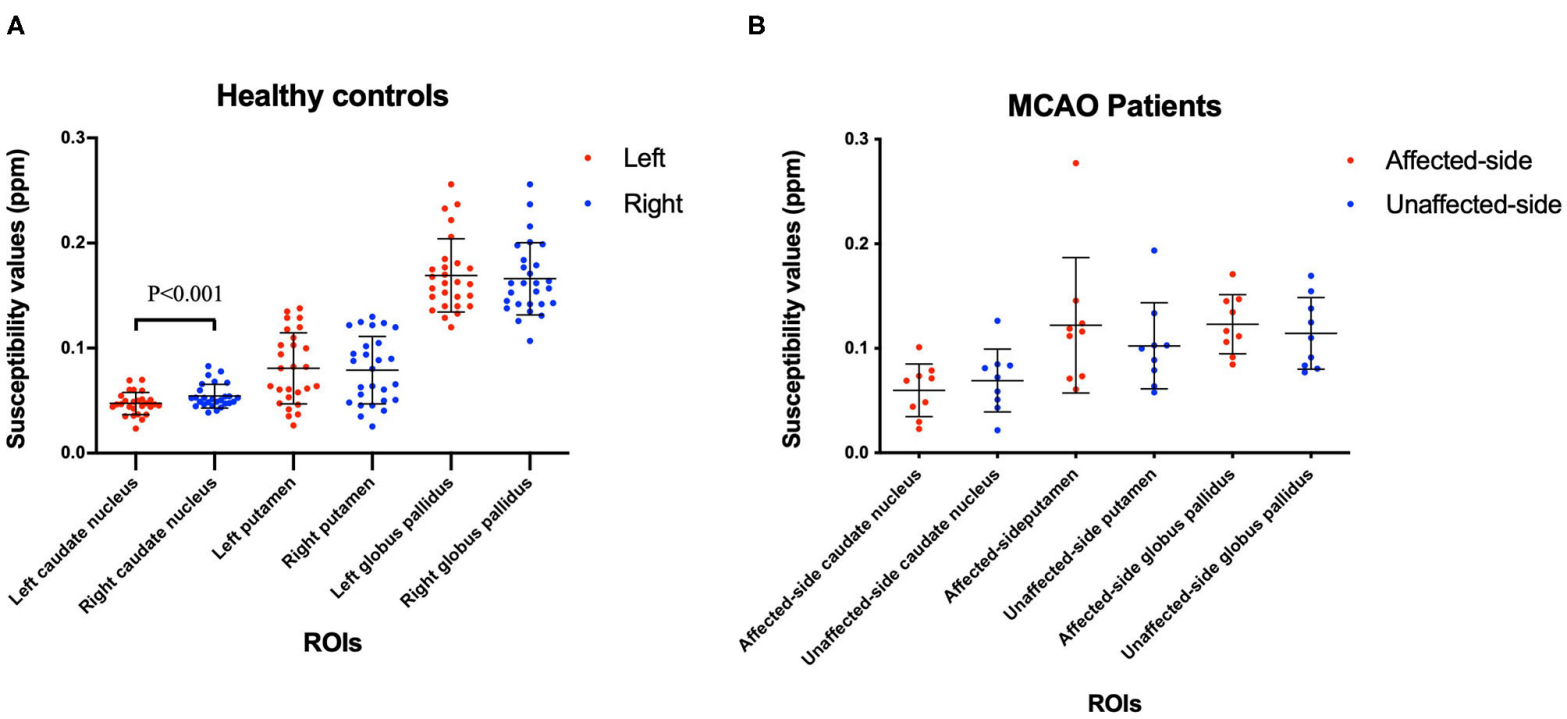
Comparisons the left and right ROIs in healthy controls **(A)**, and comparisons of the affected-side and unaffected-side ROIs in patients with MCAO **(B)**. Data was mean ± SD, *N* = 27 for healthy controls, and *N* = 9 for MCAO patients. Paired *t*-test was conducted between bilateral hemispheres for the same patient and *P* < 0.05 was considered as significant. MCAO, middle cerebral artery occlusion; ROIs, regions of interest; SD, standard deviation.

The mean and SD of the susceptibility values of the Cd, Pt, and Gp in all participants are summarized in Tables 4, 5. There were significant differences between the left and right susceptibility values of the Cd in the healthy controls, and the right was slightly higher than the left. Also, there were no significant differences between the left and right Pt and Gp in the healthy controls and no significant differences between the affected side and unaffected side in all ROI in patients with MCAO (Figure 2).

**Table 4:** Mean and SD of the susceptibility values of bilateral caudate nucleus (Cd), putamen (Pt), and globus pallidus (Gp) in all participants.

	**Caudate nucleus (Cd)**		**Putamen (Pt)**		**Globus pallidus (Gp)**	
	**Left**	**Right**	**Left**	**Right**	**Left**	**Right**
Healthy	0.0473 ± 0.01065	0.0543 ± 0.01137	0.0808 ± 0.03398	0.0791 ± 0.03210	0.1692 ± 0.03488	0.1660 ± 0.03443
MCAO	0.0660 ± 0.03143	0.0631 ± 0.02461	0.1004 ± 0.03240	0.1242 ± 0.06869	0.1188 ± 0.03535	0.1188 ± 0.02758

*SD, standard deviation; Cd, caudate nucleus; Pt, putamen; Gp, globus pallidus; MCAO, middle cerebral artery occlusion*.

**Table 5:** Mean and SD of the susceptibility values of the affected side and unaffected side caudate nucleus (Cd), putamen (Pt), and globus pallidus (Gp) in all participants.

	**Caudate nucleus (Cd)**		**Putamen (Pt)**		**Globus pallidus (Gp)**	
	**Affected side**	**Unaffected side**	**Affected side**	**Unaffected side**	**Affected side**	**Unaffected side**
MCAO	0.0599 ± 0.02532	0.0692 ± 0.03014	0.1221 ± 0.06470	0.1025 ± 0.04104	0.1232 ± 0.02827	0.1144 ± 0.03418

*SD, standard deviation; Cd, caudate nucleus; Pt, putamen; Gp, globus pallidus; MCAO, middle cerebral artery occlusion*.

### Comparisons of Susceptibility Values Between Patients With MCAO and Healthy Controls

**Figure 3:**
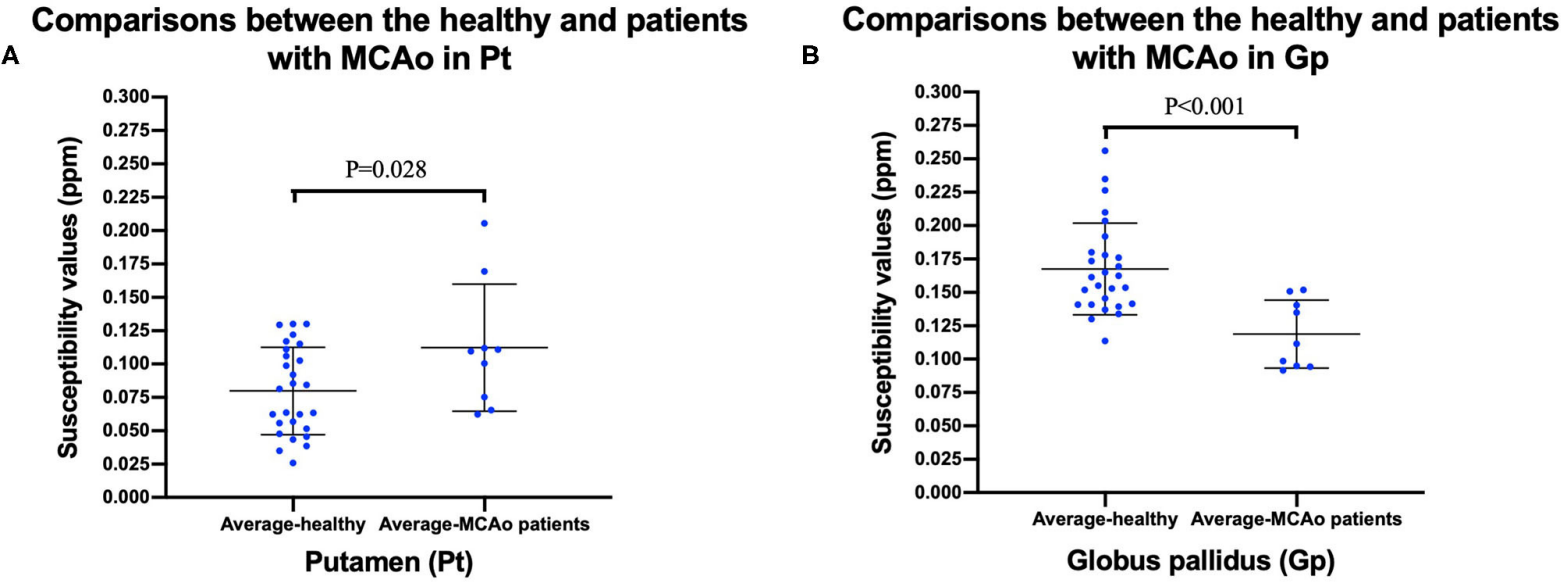
Comparisons between healthy controls and patients with MCAO in putamen (Pt) **(A)**, and globus pallidus (Gp) **(B)**. Data was mean ± SD, *N* = 27 for healthy controls, and *N* = 9 for patients with MCAO. Two sample *t*-test was conducted between two groups and *P* < 0.05 was considered as significant. MCAO, middle cerebral artery occlusion; Cd, caudate nucleus; Pt, putamen; Gp, globus pallidus; SD, standard deviation.

To facilitate the comparison between the patient group and the healthy control group, we calculated the average susceptibility values of the left and right ROI, since there was no significant difference between the left and right ROI both in MCAO patients and healthy controls, except the Cd of the patient group. There was no significant difference between the healthy group and the MCAO patient group both in the affected side and unaffected side of the Cd. Figure 3 shows the comparisons between the healthy group and the patient group in the Pt and Gp. Figure 3 shows that the susceptibility values of the Pt were significantly higher in patients with MCAO than those in the controls (*P* < 0.05), and the susceptibility values of the Gp were significantly lower in patients with MCAO than those in the controls (*P* < 0.05).

**Figure 4:**
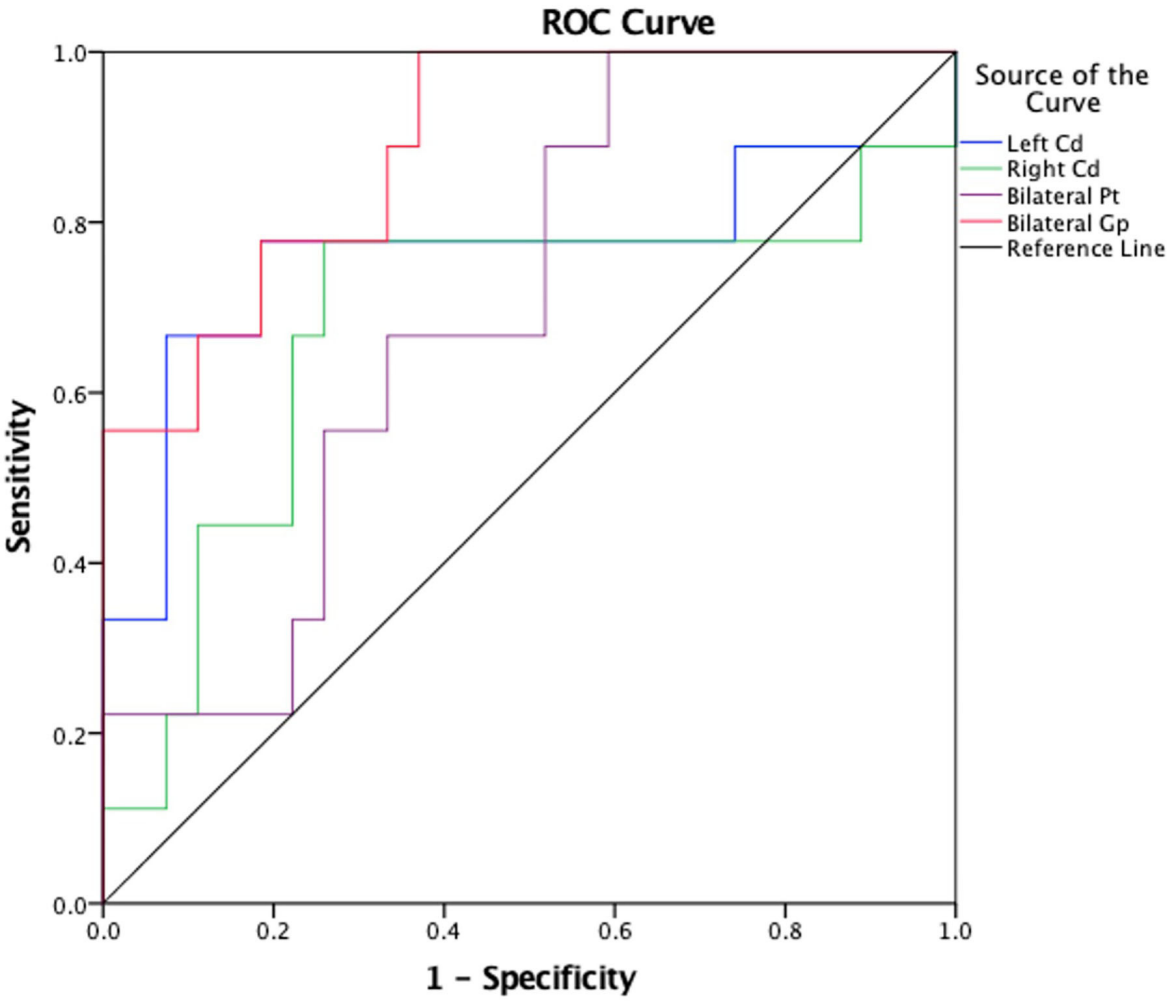
Receiver operating characteristic (ROC) curve was generated using the left Cd, right Cd, bilateral Pt, bilateral Gp for differentiating MCAO patients and healthy controls. This figure showed that the area under the curve (AUC) of average susceptibility values of the Gp (AUC = 0.889) was larger than others, including left Cd, right Cd, bilateral Pt (AUC = 0.761, 0.679, and 0.700, separately). ROC, receiver operating characteristic; Cd, caudate nucleus; Pt, putamen; Gp, globus pallidus; MCAO, middle cerebral artery occlusion; AUC, area under the curve.

The susceptibility values of the bilateral Pt in MCAO patients were significantly higher than those in the controls. Figure 4 shows that the AUC of average susceptibility values of the bilateral Gp (AUC = 0.889) was larger than the others, including the left Cd, right Cd, and bilateral Pt (AUC = 0.761, 0.679, and 0.700, respectively). The cutoff of the susceptibility value of Pt was 0.0996. The sensitivity and specificity for identifying patients with MCAO from the controls were 66.7 and 66.7%, respectively. In contrast, the susceptibility values of the bilateral Gp in patients with MCAO were significantly lower than those in the controls (*P* < 0.05). The cutoff of the susceptibility value for Gp was 0.1408. The sensitivity and specificity for differentiating patients with MCAO from the controls were 77.8 and 81.5%.

## Discussion

In the current study, susceptibility values acquired by QSM were used to investigate the alterations of the iron level in the bilateral basal ganglia region in patients with MCAO. We found some alterations of susceptibility values in patients with MCAO and some significant differences between patients with MCAO and healthy controls. More specifically, the susceptibility values of the Pt in patients with MCAO were significantly higher than those in healthy controls, and the susceptibility values of the bilateral Gp were significantly lower in patients with MCAO than in the healthy controls. In addition, the results also showed that the susceptibility values of bilateral Cd were asymmetric in healthy controls; however, the asymmetry disappeared in patients with MCAO. There were no significant differences between the affected side and the unaffected side in patients with MCAO.

The human cerebrovascular system has several compensatory neuroprotective approaches in response to ischemic attack, which help to diminish the neuronal damage. During the disease process of middle cerebral artery stenosis or occlusion, the compensatory neuroprotective mechanisms would be automatically activated if ipsilateral cerebral blood flow is insufficient and impacts normal brain function. Principal compensation approaches include the opening of communicating branches of Willis circle, the increase of vascular internal diameter, bloodstream speeding, and so on. The above compensatory reflections may contribute to the abnormal iron metabolism and consequent alterations of iron level in the bilateral basal ganglia region, which was found in the present study. However, further research is necessary to investigate it.

Iron plays a vital role in maintaining numerous fundamental biological functions of the human brain. However, disorders of iron homeostasis or excessive iron accumulation may lead to abnormal generation of toxic free radicals and consequent oxidative damage (Bolt and Marchan, 2010; Kell, 2010), which are molecular and pathological characteristics of some neurodegenerative diseases such as Alzheimer's disease, Parkinson's disease, and Huntington's disease (Ward et al., 2014; Masaldan et al., 2019; Thomas et al., 2020).

In the current study, we found that a significant increase of iron levels in the Pt was presented in patients with MCAO, indicating that MCAO could result in pathological changes of microscopic and molecular properties in the basal ganglia region. Possible interpretations for this pathological alteration are as follows: Firstly, inadequate blood flow and oxygen supply induced by MCAO can result in the proliferation, activation, and migration of astrocytes and microglia (Hu et al., 2015; Gulke et al., 2018; Zhang et al., 2019). Astrocytes and microglia significantly impact iron transport or storage mechanisms, which were involved in iron-mediated toxicity (Bishop et al., 2011). Secondly, ischemic stroke, including MCAO, can trigger a cascading pathological noninfectious neuroinflammation and the subsequent excessive release of proinflammatory cytokines, including interleukin-1 (IL-1), tumor necrosis factor alpha (TNF-α), and interleukin-6 (IL-6) (Fujioka et al., 2003; Wang et al., 2007; Chamorro et al., 2016). Neuroinflammation might affect the iron homeostasis in the CNS glia. Abnormal TNF-α release could result in increased uptake and retention of iron both in astrocytes and microglia (Rathore et al., 2012). Thirdly, we speculated that injury of the blood–brain barrier induced by ischemia attack might be another reason leading to iron deposition. It is well-recognized that the blood–brain barrier plays a crucial role in regulating the physiological transportation and metabolism of various endogenous substances and ions, including iron (Khan et al., 2018; Chiou et al., 2019; Jackson et al., 2019; Linville et al., 2019). Ischemia-related damage of endothelial cells of the blood–brain barrier significantly impairs the normal absorption of iron, and excessive amounts of iron consequently cross into the brain tissue and can be absorbed by microglia and astrocytes (McCarthy and Kosman, 2015; Simpson et al., 2015; Bu et al., 2019; Yan and Zhang, 2019), which eventually leads to increased iron level in the Pt.

Accumulating studies indicated that iron overloading and the consequent spontaneous release of neurotoxic free iron in these brain regions could result in neuronal death and memory dysfunction (Rouault, 2001). It had been demonstrated that neuronal death of the Cd and Pt could result in a series of neurocognitive dysfunctions including cognition deficit (Petty et al., 1996; Du et al., 2018). Our findings in this study provided a probable explanation regarding the neurological symptoms manifested in MCAO patients.

Interestingly, the iron level in the bilateral Gp in MCAO patients was significantly decreased when compared with that in healthy controls. Possible explanations are as follows: Firstly, a significant fluctuation of brain iron may occur during the poststroke recovery. Following the ischemic attack, various endogenous brain repair processes would be activated and engaged in functional recovery (Zhang and Chopp, 2015; Zhao and Willing, 2018). Notably, it had been demonstrated that iron metabolism was involved in the spontaneous repair in chronic ischemic brain regions (Shin et al., 2018; Kim et al., 2020). We postulated that the active iron transport and the imbalance of iron metabolism along with the brain repair process may result in a decreased iron level in some brain nucleus. Secondly, the TNF-α and interferon-γ expressed by activated microglia presented an iron-mediated toxic effect on oligodendrocytes (Zhang et al., 2005), which could trigger a subsequent release of intracellular iron (Zhang et al., 2006). The liberated iron could be detoxified and cleared from the brain region, which ultimately decreased the iron level. In addition, the decrease of blood supply and iron-rich erythrocytes after MCAO might be another reason for this phenomenon. Decreased cerebral blood flow implied a significant reduction of iron coming from erythrocytes in affected brain regions, such as the Gp.

The limitations of this study should not be neglected. First, the number of patients with MCAO enrolled in this study was limited, and the conclusions need to be verified by future studies with a larger sample size. Second, the voxel of imaging was large, and a single voxel displayed an average measurement of the neuronal environment. Third, it is hard to completely avoid the slight errors when drawing the ROIs due to the undefined nucleus boundary in current images. Therefore, further study should be conducted to thoroughly explore the potential pathophysiological roles of the basal ganglia region in patients with MCAO and the correlations between the occurrence and progression of ischemia and the alterations of the iron level in the basal ganglia region.

## Conclusions

As measured by QSM, iron levels of the bilateral basal ganglia region were significantly changed in patients with MCAO. Iron dyshomeostasis in the basal ganglia region might be involved in the pathophysiological process of middle cerebral artery stenosis and occlusion. These findings may provide a novel insight to profoundly address the pathophysiological mechanisms of MCAO.

## Data Availability Statement

The original contributions presented in the study are included in the article/supplementary materials, further inquiries can be directed to the corresponding author.

## Ethics Statement

The studies involving human participants were reviewed and approved by China-Japan Friendship Hospital. The participants provided their written informed consent to participate in this study. Written informed consent was obtained from the individuals for the publication of any potentially identifiable images or data included in this article.

## Author Contributions

LD, ZZ, and XL designed this research, analyzed the MRI data, and drafted this manuscript. LD and YC drew the regions of interest. WG, YW, JL, and BL did the MRI scanning. GM revised the whole manuscript. All authors contributed to the article and approved the submitted version.

## Conflict of Interest

The authors declare that the research was conducted in the absence of any commercial or financial relationships that could be construed as a potential conflict of interest.
